# Difficult‐to‐treat asthma patients from ethnic minority groups in central England are at an enhanced risk of house dust mite sensitisation

**DOI:** 10.1002/clt2.12303

**Published:** 2023-10-08

**Authors:** Adel H. Mansur, Julie Marsh, Ali Bahron, Maximillian Thomas, Gareth Walters, John Busby, Liam G. Heaney, Mamidipudi Thirumala Krishna

**Affiliations:** ^1^ Birmingham Regional Severe Asthma Service Birmingham Heartland Hospital University Hospitals Birmingham NHS Foundation Trust Birmingham UK; ^2^ Institute of Inflammation and Ageing University of Birmingham Birmingham UK; ^3^ Centre for Public Health School of Medicine Dentistry and Biomedical Sciences Queens University Belfast Belfast UK; ^4^ Wellcome‐Wolfson Centre for Experimental Medicine School of Medicine Dentistry and Biomedical Sciences Queen's University Belfast Belfast UK; ^5^ Institute of Immunology and Immunotherapy University of Birmingham and University Hospitals Birmingham NHS Foundation Trust Birmingham UK

**Keywords:** biomarkers, disparities, ethnicity, house dust mite, severe asthma

## Abstract

**Background:**

House dust mite (HDM) is the most common sensitising allergen in asthma. Ethnic minority groups (EMGs) in the UK are more likely to live in deprived conditionings with a greater exposure to HDM and other aero‐allergens.

**Aim:**

To compare the ethnicity‐based patterns of sensitisation to aero‐allergens and the impact of ethnicity on clinical outcomes in patients with difficult‐to‐treat asthma (DTA).

**Methods:**

Data of patients with DTA were extracted from the registry of the Birmingham Regional Severe Asthma Service (BRSAS), which have a catchment population of 7.3million from Central England. Patients from White and EMG backgrounds were compared in terms of the prevalence of atopy, total serum immunoglobulin E (IgE), specific serum IgE (ssIgE) and asthma related clinical outcomes. Logistic regression analysis was conducted to explore ethnicity‐based risk factors for HDM sensitisation.

**Results:**

A total of 1272 patients [White 1016 (79.9%), EMG 256 (20.1%) EMG] with a median age of 51 years (range 16–97) were included in the analysis. Patients from EMG were more likely (64%) to reside in the worst scale of index of multiple deprivation (IMD) than the White patients (25.5%), *p* < 0.0001. Positive HDM sensitisation was more prevalent in the EMG than in the White group [142/216 (66%) versus 375/842 (45%), *p* < 0.0001]. The median HDM ssIgE level was higher in the EMG than in the White group [3.0 KUA/L (IQR 0.06, 11.5) versus 0.1 (0.01, 3.0), *p* < 0.000001]. The odds ratio for positive sensitisation to HDM conveyed by the EMG status was 2.61 (95%CI, 1.8–3.8), *p* < 0.0001. Compared to the White group, the EMG had higher median total serum IgE [326 KU/L (115, 971) versus 114 (29.8, 434.8), *p* < 0.000001], higher blood eosinophil count (0.36 × 10^9^(0.18, 0.62) versus 0.23 (0.1,0.47), *p* < 0.000001), were marginally more atopic (79.2% vs. 75.6%, *p* = 0.098) and were less likely to being on maintenance oral corticosteroids (22% vs. 39.7%, *p* < 0.0001).

**Conclusion:**

In this DTA cohort, positive HDM sensitisation was greater amongst the EMG than the White patients. The EMG status was a significant risk factor for HDM sensitisation.

## 1 INTRODUCTION

Ethnicity‐based disparities in allergy and asthma attracted great interest in the clinical and scientific arenas in recent years. High income countries (HICs) including the UK, USA and Australia have immigrant population from the Indian subcontinent, Africa and elsewhere. There is evidence that clinical outcomes are poorer in ethnic minority groups (EMGs) in asthma, food allergy and anaphylaxis.^1^, ^2^, ^3^, ^4^ Recent evidence suggests a high prevalence of allergic diseases amongst immigrant population resident in HICs as opposed to relatively lower burden of these conditions in the native population resident in their respective countries.^5^, ^6^, ^7^, ^8^ The immune mechanisms underpinning this ‘immigrant phenomenon’ have not been elucidated.

Most evidence regarding aetio‐pathogenesis and therapeutics in allergic conditions comes from research conducted in the Caucasian White populations in HICs. House dust mite (HDM) is the most common allergen implicated globally in allergic airways disease, and environment plays a key role in sensitisation.^9^ Higher rates of sensitisation to cockroach and outdoor aeroallergens have been reported amongst American‐African children in comparison to non‐Hispanic White population in the USA and have been linked to residence in a deprived environment.^10^, ^11^ There is evidence that a significant proportion of the British EMGs live in deprived areas, which might make factors such as indoor and outdoor environment, literacy and access to healthcare less favourable from an allergy viewpoint.^12^ There are limited published data regarding ethnicity‐based disparities in allergic diseases amongst British EMGs.^13^ A recent study by Busby et al highlighted ethnicity‐based disparities in demographics, physiological parameters, T2 biomarkers and clinical outcomes in British patients with severe asthma.^1^

A clear understanding of ethnicity‐based disparities in allergen sensitisation in asthma might be relevant to primary and secondary prevention strategies and shaping better healthcare policies to address inequity and inequalities of care. There is some evidence that grass pollen and birch pollen immunotherapy prevent progression to asthma in patients with hay fever, although similar evidence is lacking in the context of HDM related allergic rhinitis.^14^ However, recent evidence has highlighted the place for HDM sublingual immunotherapy in patients with moderate‐severe asthma.^15^

The Birmingham Regional Severe Asthma Service (BRSAS) is one of the largest severe asthma centres in the UK, serving a population of 7.3 million from central England (West Midlands, Derbyshire and Gloucestershire). BRSAS catchment area is geographically wide, ethnically varied and include Birmingham which is the second largest city in the UK with a great ethnicity mix in the population (1.12 m; 37.5% from EMGs) and high deprivation index making delivery of an equitable and standardised management for severe asthma challenging,^16^ contrasted with patients from outside Birmingham who reside in towns and cities in the region that generally have lower EMG representation.^17^ BRSAS is also one of the earliest established severe asthma centres in UK^18^ making it well suited to study aeroallergen sensitisation patterns in patients attending this service from different ethnic backgrounds.

The main aim of this study was to investigate ethnicity‐based disparities in atopic status, sensitisation patterns to common aeroallergens and determine the risk factors for HDM sensitisation employing the BRSAS Registry in patients with difficult‐to‐treat asthma (DTA). Secondary aims were to compare physiological variables and clinical outcomes.

## 2 METHODS

### 2.1 Study design

This was a cross‐sectional study of patients with DTA presenting to our service in which allergic sensitisation and other outcomes were compared between the White and EMG patients.

### 2.2 Setting and participants selection

The Birmingham Regional Severe Asthma Service (BRSAS) Dendrite Clinical Systems registry collected data on all patients referred to our centre with DTA, and for this study we included data from patients whose first assessment in our service was between January 2010 – December 2021. Patients provided written consent to be included in the registry and the study is approved by the University Hospitals Birmingham NHS Teaching Trust as a service improvement project (CARMS‐19251). The sources of referrals to BRSAS were mainly from secondary care (93%) and minority from primary care (7%).^19^ The referrals to BRSAS involve DTA (asthma uncontrolled despite apparent high level of treatment in the form of high dose inhaled corticosteroid (ICS), other controller treatment with requirement for systemic corticosteroids as short burst or maintenance therapy), and 84% of the cohort had a confirmed diagnosis of severe asthma as per European Respiratory Society/American Thoracic Society guidelines.^19^, ^20^ Geographically, BRSAS covers a population of 7.3 million who reside in the West Midlands, Gloucestershire and Derbyshire with a diverse ethnic mix of higher representation of people from EMG in inner city Birmingham and a majority White population from other towns and cities in this region of the UK.

#### 2.2.1 Variables, data sources and measurements

The registry recorded data on demographics, clinical characteristics, type 2 inflammation (T2) biomarkers and phenotyping, treatment, and ethnic origin (White, South‐East Asian, Oriental, African, Mixed and Other groups). The parameters tested in this study represented baseline cross‐sectional clinical data obtained at the first patient review in the service.

Specific serum IgE (ssIgE) to aeroallergens was measured on the ImmunoCAP 250 platform (Thermo‐Fisher Scientific, UK). Atopy was defined as having a positive ssIgE result to at least one allergen (positive ssIgE ≥0.35 KUA/L). Asthma control was measured using the asthma control questionnaire (ACQ‐7)^21^ and quality of life was measured using the Euro‐QoL EQ‐5D‐5 L healthscale.^22^ Severe asthma exacerbation was defined as those requiring systemic corticosteroids treatment of ≥3 days 20 mg/day of prednisolone or double of baseline maintenance dose of prednisolone. Patients were considered adherent to inhaled corticosteroids (ICS) if their prescription ration (PPR) was ≥70% (PPR = number of ICS inhalers collected over the preceding 12 months divided by 12) The Global Lung Initiative (GLI) was used to calculate predicted lung function measures of forced expiratory volume in one second (FEV_1_), forced vital capacity (FVC), FEV_1_/FVC ratio, and the lower limit of normal (LLN) for FEV_1_, which identifies the lower 5th percentile of healthy non‐smokers.^23^

Index of Multiple Deprivation as quintile (IMD‐quintile) was calculated from patients post code (https://imd‐by‐postcode.opendatacommunities.org/imd/2019).

### 2.3 Bias and study size

To reduce the risk of selection bias, we included all patients referred and seen in our centre with data on the registry during the period from January 2010 to December 2021. This period represented an acceptable level of data quality and completeness for the whole cohort. Due to the low numbers, the non‐White patients were grouped together into a single category of ethnic minority group (EMG) to improve the power of the study.

### 2.4 Statistical methods

Comparisons between White and EMG were conducted using parametric and non‐parametric tests as deemed appropriate. Sensitisation to HDM and other aeroallergens were compared both as dichotomous and continuous variables, using chi‐squared tests (categorical data), Kruskal Wallis tests (non‐parametric continuous data) and one way analysis of variance (ANOVA) (ordinal data). Normal distribution of data was assessed by D’Agostino‐Pearson test. Multivariate logistic regression was conducted to investigate the effect of ethnicity, IMD‐quintile, age, gender, and blood eosinophils on HDM sensitisation (defined as ssIgE ≥0.35 KUA/L). Goodness of fit was examined using the Hosmer‐Lemeshow test. Lung function, biomarkers, asthma control and exacerbations were compared as continuous or binary variables as appropriate. Statistical analysis was conducted using MedCalc® Statistical Software version 20.114 (MedCalc Software Ltd, Ostend, Belgium; https://www.medcalc.org; 2022).

## 3 RESULTS

### 3.1 Study population demographics

The clinical characteristics of this study population at the time of the first assessment at our service are provided in Table 1. A total of 1272 patients comprised of 1016 (79.9%) White (Caucasian) and 256 (20.1%) EMG [South Asian 200 (15.7%), Black 14 (1.1%), Oriental 1 (0.08%), mixed 11 (0.9%), any other 30 (2.4%)] were included in the analysis. The total cohort median age was 51 (IQR 38, 61) years (range 16–97). The median age of the White group was higher than the EMG [52 (39, 62) versus 47.5 (36, 58) years; p 0.0003], but the age at the onset of asthma symptoms was similar. The body mass index (BMI) was significantly higher in the White [31 kg/m^2^ (25.9, 36.3)] than the EMG [29.2 (24.8, 34.7) alongside observed excess ex‐smokers in the White group. However, the IMD was significantly worse in the EMG than the White group in which 64% of the EMG fell in the worse scale of IMD compared to 25.5% of the White group (*p* < 0.0001). The EMG had a significantly lower median FEV_1_ than the White group [pre‐bronchodilator FEV_1_% predicted; EMG 67.5% versus White 72.9%, *p* = 0.0016], with 67.6% of the EMG had FEV_1_ < LLN compared to 56.2% of the White group, *p* = 0.0083. EMG also had lower FVC and lower total lung capacity (TLC) than the White group, but had similar residual volume and coefficient diffusion of carbon monoxide (KCO).

**TABLE 1:** Demographics and clinical characteristics of White and EMG patients presenting to a severe asthma service.

	Total	White	EMG	*p*‐value
Number	1272	1016	256	0.0003
Median age years	51 (38,61)	52 (39,62)	47.5 (36,58)	
Number	1123	900	223	0.28
Age of onset of symptoms (years)	16 (4.0,36.5)	16.0 (4,36.5)	15.0 (4.3,31)	
Number	1272	1016	256	0.059
Females (%)	896 (70.4%)	728 (71.7%)	168 (65.6%)	
Number	1233	992	241	0.0008
BMI kg/m^2^ median (IQR)	30.7 (25.6,36)	31 (25.9,36.3)	29.2 (24.8, 34.7)	
Number	1272			N/A
Ethnic origin		1016 (79.9%)	256 (20.1%)	
White	1016 (79.9%)			
South Asian	200 (15.7%)			
Oriental	1 (0.08%)			
Black	2 14 (1.1%)			
Mixed	11 (0.9%)			
	30 (2.4%)			
Number	1219	966	253	<0.0001
IMD quintile score				
1 (least deprived)	172 (14.1%)	156 (16.1%)	16 (6.3%)	
2	201 (16.5%)	189 (19.7%)	12 (4.7%)	
3	229 (18.8%)	206 (21.3%)	23 (9.1%)	
4	209 (17.1%)	169 (17.5%)	40 (15.8%)	
5 (most deprived)	408 (33.5%)	246 (25.5%)	162 (64.0%)	
Number	1257	1016	256	<0.0001
Never smoked	820 (65.2%)	625 (62.2%)	195 (77.1%)	
Ex‐smoker	350 (27.8%)	310 (30.9%)	40 (15.8%)	
Current smoker	87 (6.9%)	69 (6.9%)	18 (7.1%)	
Number	1101	876	225	
Pack years median (IQR)	0.0 (0.0,5.0)	0.0 (0.0,5.0)	0.0 (0.0,0.0)	0.000001
Number	1200	953	243	0.0003
Pre‐bronchodilator FEV_1_ (L)	2.04 (1.48,2.7)	2.11 (1.5,2.8)	1.88 (1.39,2.4)	
<LLN	691/1190 (58.1%)	533/949 (56.2%)	158/241 (65.6%)	0.0083
Number	1185	943	239	0.0016
Pre FEV1% predicted	71.2 (53.3, 89.5)	72.9(53.9,92)	67.5 (52.3,80.1)	
Number	650	537	113	0.03
Post‐bronchodilator FEV_1_ (L)	2.26 (1.8,2.9)	2.3 (1.8, 2.9)	2.1 (1.62,2.7)	
Number	1187	948	239	<0.000001
Pre‐bronchodilator FVC (L) FVC % predicted	3.0 (2.4,3.8)	3.12 (2.5,3.9)	2.7 (1.0,3.3)	
Number	1176	938	234	<0.000001
Pre FVC % predicted	86.4 (71.4, 99.6)	88.5 (73.7, 101.8)	78.9 (63.8, 92.4)	
Number	632	522	110	0.00003
Post‐bronchodilator FVC (L)	3.3 (2.7, 4.06)	3.37 (2.9,4.1)	2.9 (2.38,3.8)	
Number	1187	948	239	0.004
Pre‐bronchodilator FEV1/FVC ratio	0.69 (0.57,0.79)	0.67 (0.56,0.78)	0.71 (0.6,0.8)	
Number	457	392	65	<0.000001
Total lung capacity %pred	95 (84,106)	97 (86,106)	85 (56,96)	
Number	438	372	66	0.09
Residual volume %pred	96.5 (76,122)	98 (77,123)	88.5 (73,114)	
Number	553	469	84	0.38
Diffusion coefficient (KCO) %predicted	101 (90,113)	101 (90,112)	104 (91,114)	
Number	1097	889	208	0.8
ACQ‐7 (0–6 scale)	3.0 (1.3,3.9)	3.0 (1.3, 3.9)	2.9 (0.8,4.0)	
Number	1008	844	164	0.004
EuroQoL health scale (0–100)	50 (35,70)	50 (35,70)	45 (30,70)	
Number	1027	811	216	0.61
Severe exacerbations/last year	5.0 (3.0,8.0)	5.0 (2.0,8.0)	5.0 (3.0,8.0)	
Number	1245	995	250	<0.0001
On maintenance OCS (yes)	450 (36.1%)	395 (39.7%)	55 (22.0%)	
Number	1244	992	252	0.14
Inhaled corticosteroids (yes)	1188 (95.5%)	943 (95.1%)	245 (97.2%)	
Number	1136	898	238	0.7
ICS (BDP equivalent mg/day)	2.0 (1.0,2.0)	2.0 (1.0,2.0)	2.0 (1.0,2.0)	
Number	225	162	63	<0.001
ICS Non‐adherence PPR <70%	66 (29.3%)	43 (26.5%)	23 (36.5%)	
Number	1244	992	252	0.15
LABA (yes)	1109 (89.1%)	878 (88.5%)	231 (91.7%)	
Number	1129	893	236	0.12
LAMA (yes)	534 (47.3%)	433 (48.5%)	101 (42.8%)	
Number	1243	992	251	0.11
LTRA (yes)	824 (66.3%)	647 (65.2%)	177 (70.5%)	
Number	1252	998	254	0.89
Theophylline (yes)	478 (38.2%)	382 (38.3%)	96 (37.8%)	
Number	986	779	207	0.5
Biologic therapy at point of referral (yes)	25 (2.5%)	21 (2.7%)	4 (1.9%)	
Number	903	729	174	0.05
Emergency department visits/last year	5.0 (2.0,10.0)	5.0 (2.0,10.0)	5.5 (3.0,10.0)	
Number	1175	946	229	0.7
Hospitalisation last year	1.0 (0.0,2.0)	1.0 (0.0,2.0)	1.0 (0.0,2.0)	
Number	1129	897	232	<0.000001
Median eosinophils x10^9^/L	0.26 (0.11,0.51)	0.23 (0.1,0.47)	0.36 (0.18,0.62)	
Number	992	802	190	0.15
FeNO ppb	28 (13,56)	27 (13,56)	30.5 (15,57)	
Number	1238	988	250	0.019
Allergic rhinitis (yes)	375 (30.3%)	284 (28.8%)	91 (36.4%)	
Number	1170	935	235	0.6
Nasal polyps (yes)	166 (14.2%)	135 (14.4%)	31 (13.2%)	
Number	1157	922	235	0.14
Atopic dermatitis (yes)	316 (27.3%)	247 (26.8%)	69 (29.4%)	
Number	1069	859	210	0.27
NSAID intolerance (yes)	158 (14.9%)	132 (15.4%)	26 (12.4%)	
Number	1195	952	243	0.85
Occupational worsening of asthma (yes)	119 (10.0%)	94 (9.9%)	25 (10.3%)	

*Note*: Table provides details of the cross sectional data at the point of first assessment to our severe asthma service. Data are presented as median (IQR) for continuous variables proportions/percentages for binary variables unless stated otherwise.

Abbreviations: ACQ, asthma control questionnaire; BDP, beclomethasone equivalent; BMI, body mass index; EuroQoL, European quality of life questionnaire; FeNO ppb, fractional exhaled nitric oxide in particles per billion; FEV1, forced expiratory volume in 1 second; FVC, forced vital capacity; ICS, inhaled corticosteroids; IMD, index of multiple deprivation score; IQR, interquartile; LABA, long acting β2‐agonist; LAMA, long acting muscarinic antagonist; LTRA, leukotriene receptor antagonist; NSAID, non‐steroidal anti‐inflammatory drug; OCS, oral corticosteroids; PPR, prescription possession ratio.

The median blood eosinophil count was significantly higher in the EMG than the White group (0.36 × 10^9^ vs. 0.23, *p* < 0.000001). The highest ever blood eosinophil count was also significantly higher in the EMG than in the White group [0.69 × 10^9^ versus 0.43 respectively, *p* < 0.000001]. However, we observed no significant difference in fraction exhaled nitric oxide (FeNO) level between the two groups. Allergic rhinitis was more prevalent in the EMG than the White group [91/250 (36.4%) versus 284/988 (28.9%), chi‐square 5.5, df 1, p 0.019]. Otherwise, we observed no significant difference in the prevalence of nasal polyps, atopic dermatitis, non‐steroid anti‐inflammatory drug (NSAID) intolerance, or occupational factors between the groups.

The ACQ‐7 was similar between the two groups. Quality of life measured by EuroQoL (EQ‐5D‐5 L) health scale was lower in EMG than the White groups [“worst” 0–100 “best” scale, 45 (30, 70) versus 50 (35, 70), *p* = 0.004].^23^ Severe exacerbations frequency and hospital admissions in the preceding year were similar between the two groups, but emergency department visits for acute asthma attacks were marginally higher in EMG than in White [5.5 (3.0, 10.0) versus 5 (2.0, 10.0), *p* = 0.05].

The proportions of patients prescribed ICS, and the median ICS dose were similar in the White and EMG groups, alongside similar prescribing patterns of long acting β2 agonist (LABA), long‐acting muscarinic antagonist (LAMA), leukotriene receptor antagonist (LTRA) and oral theophylline. In a smaller number of patients with available ICS adherence data (non‐adherence represented <70% ICS prescription possession ratio), non‐adherence was observed in 36.5% of the EMG compared to 26.5% in the White group (*p* < 0.001). The proportion of patients on oral corticosteroids (OCS) was significantly higher in the White group (39.7%) than EMG (22%), *p* < 0.0001. The number of patients receiving biologic treatment at the point of the first assessment was small (2.5%) and was not different between the two groups.

### 3.2 Atopy, total IgE and ssIgE to common aero‐allergens in White and EMG

Results of atopic trait analyses comparing EMG and White groups are provided in Table 2. The prevalence of atopy was marginally higher in the EMG (75.6%) than in the White group (70.2%), but it did not reach statistical significance. The median total serum IgE was significantly higher in the EMG than the White group [326 (115, 971) ng/L versus 114 (29.8, 434.8), *p* < 0.000001]. In addition, using total serum IgE as binary trait (high IgE ≥120 ng/L), higher IgE was observed in 74% of the EMG compared to 49% in the White group (*p* < 0.0001). The median HDM ssIgE was significantly higher in EMG than in the White group [3.0 (0.06, 11.2) KUA/L versus 1.0 (0.01, 3.0), respectively; *p* < 0.000001]. The binary HDM ssIgE trait (positive HDM ssIgE ≥0.35 KUA/L) demonstrated significantly higher sensitisation amongst the EMG (65.7%) than in the White group (44.5%), *p* < 0.0001. The median ssIgE to aspergillus *fumigatus* was higher in the EMG than the White group [0.03 (0.0, 0.3) versus 0.01 (0.0, 0.14), *p* = 0.015], however, despite a marginal increase in the binary positive aspergillus ssIgE in the EMG (24.2%), than the White group (20.9%), it did not reach statistical significance (*p* = 0.3). Similarly, the median mixed grass ssIgE was higher in the EMG than in the White group [0.15 (0.0, 3.0) versus 0.03 (0.0, 2.14), *p* = 0.03], but positive mixed grass ssIgE binary was not different between the EMG (45.3%) and the White group (41.5%), *p* = 0.3. Conversely, there was statistically non‐significant trend towards higher cat ssIgE positive binary in the White group (34%) than EMG (28.7%), *p* = 0.14, but the median cat ssIgE level was not different between the two groups. The positive dog ssIgE binary was also higher in the White 24.2% than in the EMG 20.9%, *p* = 0.05, but the median ssIgE level was not statistically different between the two groups. Exposure to pets at home was significantly higher in the White group (52.7%) than EMG group (24.8%), *p* < 0.0001. The proportion of polysensitised patients (positive to ≥2 allergens) was marginally higher in the EMG than in the White group [114/232 (49.14%) versus 383/898 (42.7%)], which did not reach statistical significance *p* = 0.08.

**TABLE 2:** Comparison of atopy, total serum IgE and specific serum IgE to common aeroallergens between the White group and EMG.

	Total	White	EMG	*p*‐value
Number	1176	942	234	0.098
Atopy positive (%)	838 (71.3%)	661 (70.2%)	177 (75.6%)	
Number	1073	865	208	<0.000001
Median (IQR) total IgE KU/L	148 (37,518.8)	114 (29.8,434.8)	326 (115,971)	
Total serum IgE binary (≥120 KU/L)	578 (53.9%)	424 (49%)	154 (74%)	<0.0001
Number	1058	842	216	<0.000001
Median ssIgE HDM KUA/L	0.26 (0.01,3.4)	0.1 (0.01,3.0)	3.0 (0.06,11.2)	
Binary ssIgE HDM (≥0.35 KUA/L)	517 (48.9%)	375 (44.5%)	142 (65.7%)	<0.0001
Number	1068	852	216	0.6
Median ssIgE cat KUA/L	0.0 (0.0,2.0)	0.0 (0.0,2.0)	0.01 (0.0,1.0)	
Binary ssIgE cat (≥0.35 KUA/L)	352 (33%)	290 (34%)	62 (28.7%)	0.14
Number	1037	820	217	0.7
Median ssIgE dog KUA/L	0.03 (0.0,1.97)	0.03 (0.0,2.0)	0.05 (0.0,0.72)	
Binary ssIgE dog (≥0.35 KUA/L)	359 (34.6%)	296 (36.1%)	63 (29%)	0.05
Number	886	700	186	0.015
Median ssIgE *asp. f.* KUA/L	0.01 (0.0,0.17)	0.01 (0.0,0.14)	0.03 (0.0,0.3)	
Binary ssIgE *asp. f.* (≥0.35 KUA/L)	191 (21.6%)	146 (20.9%)	45 (24.2%)	0.3
Number	1012	808	203	0.03
Median ssIgE grass mix KUA/L	0.06 (0.0,2.65)	0.05 (0.0,2.14)	0.15 (0.0,3.0)	
Binary ssIgE mixed grass (≥0.35 KUA/L)	427 (42.2%)	335 (41.5%)	92 (45.3%)	0.3
Number	1130	898	232	0.1
Median number of positive allergens	1.0 (0.0,3.0)	1.0 (0.0,3.0)	1.0 (0.0,3.0)	
Polysensitised (≥2 aero‐allergens), yes (%)	497 (44%)	383 (42.7%)	114 (49.1%)	0.08
Number	1047	841	165	<0.0001
Exposure to pets at home yes (%)	484 (46.2%)	443 (52.7%)	41 (24.8%)	

*Note*: Data provided as median (IQR) and as binary traits. Atopy was defined as positive specific serum IgE (ssIgE) to one or more aero‐allergens (atopic if ssIgE ≥0.35 KUA/L).

Abbreviation: *asp. f.*, *aspergillus fumigatus*.

### 3.3 Extent and determinants of HDM ssIgE sensitisation in the EMG and the White group

**FIGURE 1:**
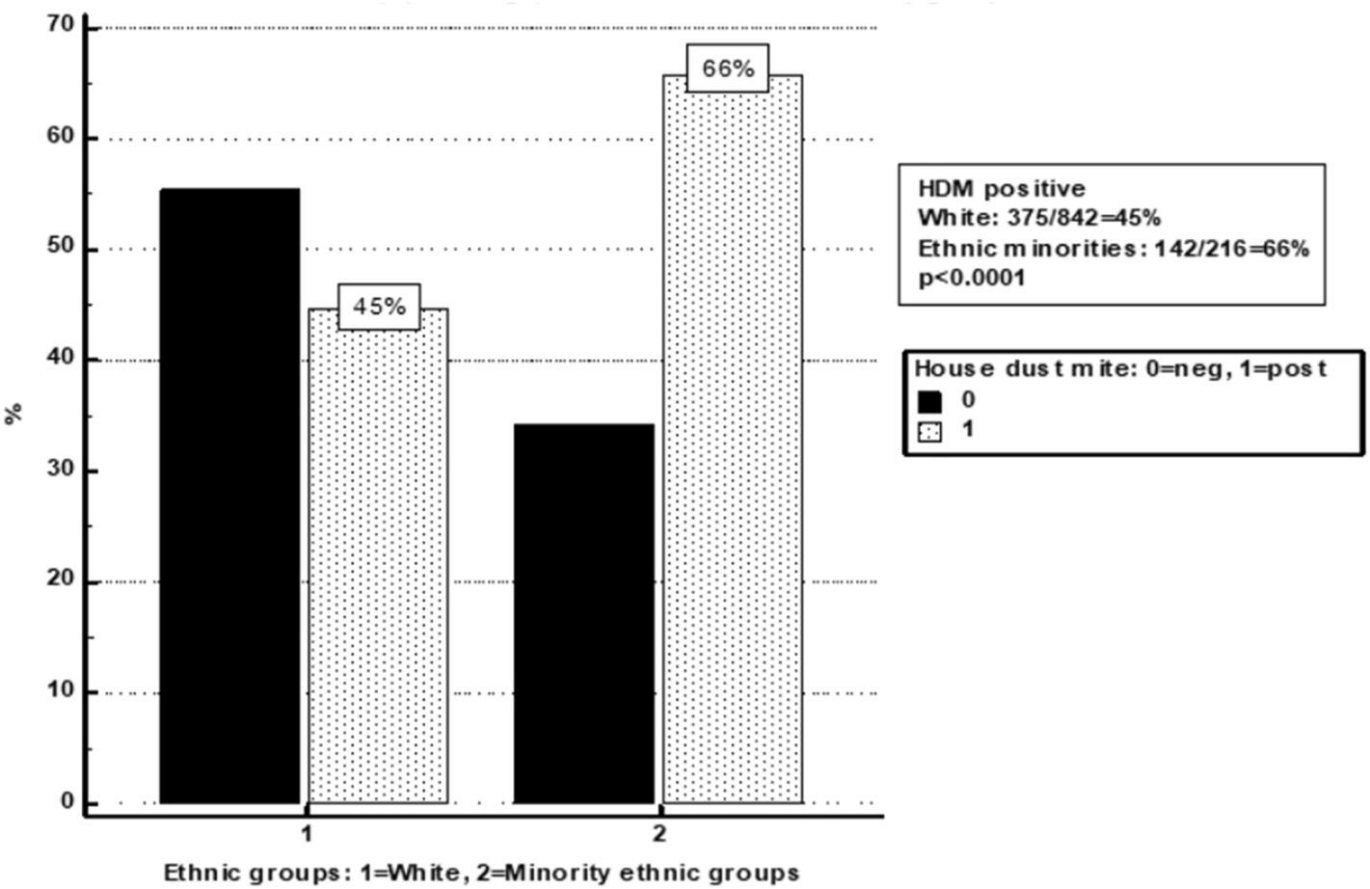
Comparisons of prevalence of positive serum specific IgE to house dust mite in EMG and White groups.

**FIGURE 2:**
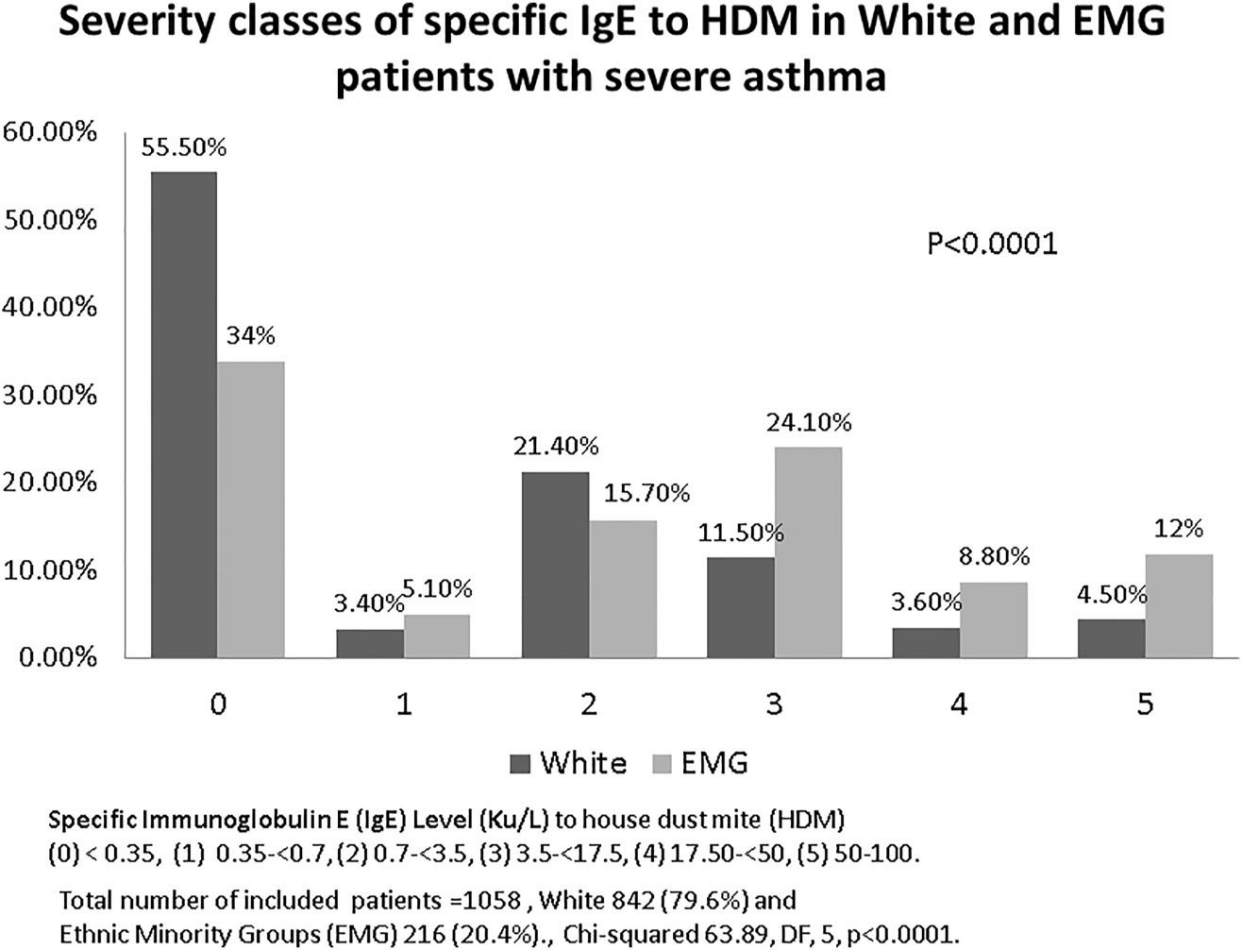
Illustration of the severity of sensitisation to HDM as measured by serum specific IgE (ssIgE) comparing the White Group and EMG. Depending on the serum level of IgE cases were divided on 6 categories. The figure demonstrates statistically significant increase in the proportion of patients from the EMG than the White in the most severe HDM sensitisation categories. Abbreviations: EMG: ethnic minority group; HDM; house dust mite, IgE; immunoglobulin E.

In addition to the significantly higher proportion of patients in the EMG group who met the positive HDM ssIgE criteria of ≥0.35 KUA/L, as compared to the White group (Figure 1), the severity, as per ‘classes’ (Class 0 ‐ Class 5) of HDM ssIgE sensitisation levels also demonstrated significantly higher representation of the EMG in the most severe sensitisation classes than the White group, thus revealing a picture of a higher overall and more severe HDM sensitisation pattern in the EMG (Figure 2).

In a logistic regression analysis model of ‘HDM binary’ as the dependent variable and ethnicity binary, IMD‐quintile, gender, age, and blood eosinophil count as independent variables, 965 cases were included (HDM positive 477 (49.4%) and HDM negative 488 (50.6%). The odds ratio (OR) for EMG as a predictor for HDM sensitisation was 2.61 [(95% CI 1.8 to 3.75); *p* < 0.0001]. This model correctly identified 64.25% of cases. The ORs for IMD‐quintiles, age, gender and blood eosinophil count were 0.89, 0.96, 0.54, and 1.0, respectively (Table 3). The inclusions of other independent factors such as BMI, QoL, FeNO, and mOCS use in the regression model were negative.

**TABLE 3:** Logistic regression analysis using the dependent variable HDM sensitisation as a binary (positive/negative HDM ssIgE).

Variable	Coefficient	Std. Error	Wald	Odds ratio	95% CI	*p*‐value
Ethnicity binary	0.96	0.19	26.8	2.61	1.8 to 3.75	<0.0001
IMD‐quintile	−0.12	0.05	5.8	0.89	0.8 to 0.98	0.0164
Age	−0.04	0.005	65.4	0.96	0.95 to 0.97	<0.0001
Gender	−0.62	0.15	16.4	0.53	0.4 to 0.73	0.0001
Eosinophil count	0.04	0.18	0.04	1.03	0.73 to 1.5	0.84

*Note*: Stepwise logistic regression analysis for the dependent variable HDM ssIgE sensitisation as a binary trait (positive sensitisation ≥35KUA/L) and the independent variables ethnicity, IMD‐quintiles, age, gender and blood eosinophil count. The sample size was 965 cases (477 HDM positive and 488 HDM negative). The overall model fit Chi‐squared 116.7, degree of freedom 5, *p* < 0.0001.

Abbreviations: IMD‐quintile, index of multiple deprivation scale from 0 to 5; Std. Error, standard error.

## 4 DISCUSSION

This is the first British study to show that ethnic minority status enhances risk for HDM sensitisation in DTA. Furthermore, our data showed greater prevalence of HDM sensitisation, higher levels of specific serum IgE to HDM with a greater proportion of EMG patients showing more severe sensitisation. Our data also showed a significantly higher total serum IgE level and peripheral blood eosinophil count, lower FEV_1_ and more frequent attendance to emergency department for acute exacerbations amongst the EMG, in keeping with recently published data^1^ from the UK.

Ethnicity‐based disparities in immune‐mediated disorders including asthma have been reported in HICs including the USA, Australia and the UK.^7^, ^13^, ^24^ In a longitudinal cohort study involving over 6 million patients in primary care spanning a decade, the incident risk of asthma, allergic rhinitis and atopic eczema was shown to be greater amongst British South Asian and Afro‐Caribbean patients than the White patients.^25^ The risk of HDM and cockroach sensitisation was found to be significantly greater amongst African‐American children in USA.^10^, ^11^ Furthermore, the risk of cockroach sensitisation was higher amongst residents in urban areas and those from lower socio‐economic strata.^10^ Interestingly, our data showed a higher prevalence of (at the standard ‘cut off’ of 0.35 KUA/L) and stronger sensitisation to HDM (45% vs. 20% in class 3–5 for specific IgE levels) in the EMG, but not to other aeroallergens including pollens, aspergillus, cat and dog, although the absolute levels of grass pollen and aspergillus specific IgE were higher in the EMG, in keeping with higher total serum IgE. There was also the observation of marginal or non‐significant trend towards higher cat and dog sensitisation level in White than in EMG patients with higher pet ownership in the White patients. The clinical significance of this later observation is uncertain and requires further study. Cockroach sensitisation was not included in the standard diagnostic workup, as it is not a common sensitising allergen in the UK and Europe. Logistic regression analysis showed that EMG status enhances the risk of HDM sensitisation by ∼2.6 fold, although this was independent of IMD. The EMG, however, were significantly more deprived (and most deprived) than the White patients. IMD is an imperfect measure of the socioeconomic status, as it is only based on the individual local area of residence and does not factor in individual circumstances including indoor and outdoor environmental allergen exposure and pollution levels, the two important determinants relevant to sensitisation.^26^ The magnitude of HDM exposure has been related to the extent of sensitisation, which was reported to be higher in poor housing due to dampness or poor ventilations.^27^ Furthermore, this study did not account for gestational exposure and early life exposure to allergens. Hence, it is not possible to categorically exclude the lack of association between deprived status and enhanced risk of HDM sensitisation. Genetic variations may also play a part in the observed ethnicity‐based differences in HDM sensitisation.^28^

Serum total IgE and peripheral blood eosinophil count are important biomarkers in allergy and asthma. The clinical significance and reasons underpinning higher levels in EMGs remain uncertain and are beyond the scope of this study. It is plausible that higher levels of T2 biomarkers including blood eosinophils, specific IgE to HDM and total IgE might at least in part be contributing to disease severity. A limitation of this analysis is that the data was not corrected and normalised for ethnicity, as ethnicity‐specific reference ranges have not yet been established and are not routinely reported in the UK NHS laboratories and variables potentially driving total IgE and peripheral blood eosinophil count were not explored and factored into the analysis. Ethnicity‐based differences in blood eosinophils, neutrophils and total serum IgE have been reported in African‐American, Mexican American and Puerto Rican children with severe asthma.^29^ This might have important implications for patient selection and effectiveness of biologic therapies used in severe asthma in EMG, such as anti‐IgE therapy (omalizumab) and anti‐interleukin (IL)‐5 agents. The current guidelines do not factor in ethnicity in patient selection,^29^ which argue for developing ethnicity‐tailored guidelines for biologic therapies in severe asthma in order to maintain equity. There is also a need to conduct further research into the impact of such differences in T2 marker levels between EMG and White groups on the overall effectiveness of biologics therapy.

The ‘actual’ FEV_1_ in the EMG was significantly lower than in the White group. The reason underpinning this observation is uncertain and is likely to be multifactorial. In part, this may be explained by previous studies reporting higher normalised FEV_1_ and FVC in the White people compared to South Asians and Black ethnicity.^30^, ^31^ However, the ‘predicted’ FEV_1_ was also significantly lower in the EMG compared to White patients. This suggests greater disease severity in the EMG. The RASP biomarker study reported that patients from EMG are more likely not to follow medical advice required to optimise asthma therapy.^32^ There is some evidence that physicians might underestimate the severity of disease amongst African‐American patients.^33^ Our data showed significantly more frequent visits to emergency units with acute exacerbations but no difference in hospitalisation rates, largely in keeping with a recent report by Busby et al.^1^ Greater acute exacerbations, hospital visits and higher rates of fatal asthma have also been reported amongst African‐American patients.^4^, ^34^ Adherence‐related issues exist amongst EMGs resident in HICs, as well as in their own native environment with a preference to oral medications (with a taboo towards inhalers) and/or to unproven complimentary therapies and may be influenced by cultural and religious factors and beliefs.^7^, ^34^, ^35^, ^36^, ^37^, ^38^, ^39^ Our data showed that there was less use of ICS amongst EMG, although maintenance OCS use was significantly greater amongst the White group. It is not known if asthma is intrinsically more severe in the EMG. A vast majority of current knowledge regarding phenotypes and clusters has been generated from research conducted amongst White patients in HICs.

The greater prevalence of HDM sensitisation in the EMG is an important finding. Early childhood HDM sensitisation was associated with the onset of asthma at the age of 11 years.^27^ HDM sensitisation was also associated with impaired anti‐viral and anti‐bacterial immunity which play a role in the pathogenesis of asthma exacerbations and asthma severity.^40^ There is inconsistent evidence that reduction in HDM exposure sensitised patients improves asthma outcomes.^41^ There is also some evidence regarding potential role for HDM sublingual immunotherapy in mild‐moderate asthma.^15^ HDM sublingual immunotherapy may have a potential role as a primary prevention strategy for children with HDM related allergic rhinitis, although evidence thus far has not been convincing and poor study design and small sample sizes have been highlighted.^14^

Blood eosinophils and total serum IgE were significantly higher in the EMG than the White group. Causes of these observations are unclear at present. Our study did not factor in diurnal variations in blood eosinophils and variation in eosinophil counts over time, however the highest‐ever eosinophil counts were also higher in the EMG than the White group. Other potential causes of eosinophilia and high total IgE that were not factored in our study include travel history, helminth infection, tuberculosis history and birthplace. A recent British study involving a pooled analysis of total white cell counts and differential counts from healthy volunteers from 35 clinical trials showed no significant differences between White and Asian group, but the total white cell count and lymphocyte counts were significantly lower amongst Black volunteers in comparison to non‐Black volunteers.^42^

This study has limitations. The EMG sample size was relatively small but proportionate to the British population demographics, and was mainly represented by patients from the Indian subcontinent with less representation of other ethnic groups. Therefore, potential differences within the EMG in allergen sensitisation patterns were not studied. Conversely, one of our study cohort strength is its wide catchment area in central England with a diverse ethnic mix. In this study, we did not investigate other important determinants of healthcare disparities such as English language proficiency, and social and religious factors.^34^ Whilst these might account for differences in asthma control and severity, they are unlikely to explain the higher prevalence of HDM sensitisation. The study was also limited by its cross‐sectional design, in which the analysis of the incidence of sensitisation events, the effect of duration and magnitude of allergens exposure on sensitisation and the fluctuating nature of parameters such as blood eosinophils and serum IgE could not be performed. Effect of helminth infection, travel history, birth place and first versus second generation immigrant status on sensitisation patterns were also not studied. The index of multiple deprivation used in this study relies primarily on post code to determine the individual's deprivation status and is not an accurate measure of indoor and outdoor pollution and allergen exposure levels. Study of housing conditions including indoor and outdoor exposures is therefore required to investigate the causes of this observed increase of HDM sensitisation in EMG.

In conclusion, our data showed that ethnic minority status enhances risk for HDM sensitisation independent of IMD in DTA and that there is a higher prevalence and severity of HDM sensitisation in the EMG alongside higher serum total IgE and blood eosinophils. Further studies are needed to investigate plausible mechanisms that enhance the risk of HDM sensitisation in EMG in order to pave the way for novel primary and secondary prevention strategies.

## AUTHOR CONTRIBUTIONS

**Adel H. Mansur**: Conceptualization (lead); data curation (lead); formal analysis (lead); investigation (equal); methodology (equal); writing – original draft (lead). **Julie Marsh**: Data curation (equal); project administration (equal); resources (equal); software (equal). **Ali Bahron**: Data curation (equal); formal analysis (equal); resources (equal); software (equal); validation (equal); writing – review & editing (equal). **Maximillian Thomas**: Data curation (equal); investigation (equal); writing – review & editing (equal). **Gareth Walters**: Conceptualization (equal); investigation (equal); methodology (equal); writing – review & editing (equal). **John Busby**: Data curation (equal); formal analysis (equal); writing – review & editing (equal). **Liam G. Heaney**: Methodology (equal); visualization (equal); writing – review & editing (equal). **Mamidipudi Thirumala Krishna**: Conceptualization (equal); data curation (equal); formal analysis (equal); methodology (equal).

## CONFLICT OF INTEREST STATEMENT

This study is an investigator led and did not receive funds from pharmaceutical companies. AHM declares institutional and personal funds for talks, advisory boards and research grants from AZ, GSK, BI, Chiesi, Teva, Novartis, Sanofi outside the submitted work. LGH declares personal fees from Novartis, Hoffman la Roche/Genetech Inc, Sanofi, GSK, AZ, Teva, Theravance, Circassia, and grants from Medimmune, Novartis UK, Roche/Genentech Inc, GSK, Amgen, AZ, Aerocrine, Vitalograph, all outside the submitted work. MTK received research grants from NIHR, MRC CiC, FSA, GCRF and University of Birmingham outside of the work presented in this manuscript. MTK is participating in a Delphi Advisory panel for treatment pathway for sublingual immunotherapy in allergic rhinitis and asthma organised by ALK Abello. MTKs department received educational grants from ALK Abello, Thermo‐Fisher Scientific, MEDA and other pharmaceutical companies for annual PracticAllergy course over the years. JB has received research grants from AstraZeneca outside the presented work and has received personal fees for advisory board attendance from NuvoAir.

## GUARANTOR STATEMENT

Professor Adel H Mansur is the guarantor of the content of the manuscript, including the data analysis.

## Data Availability

Data available on request from the authors.
